# “I still have not mastered that skill!” Medical student perspectives on a simulation-based evidence-based medicine competency assessment

**DOI:** 10.5195/jmla.2025.2023

**Published:** 2025-04-18

**Authors:** Joey Nicholson, Caitlin Plovnick, Juliana Magro, Cees van der Vleuten, Anique de Bruin, Adina Kalet

**Affiliations:** 1 Joseph.Nicholson@med.nyu.edu, Chair and Director, NYU Health Sciences Library, NYU Grossman School of Medicine, NYU Langone Health, NY; 2 caitlin.plovnick@nyulangone.org, Assistant curator and lead of education and curriculum integration, NYU Health Sciences Library, NYU Grossman School of Medicine, NYU Langone Health, New York, NY; 3 juliana.magro@nyulangone.org, Assistant curator and education and research librarian, NYU Health Sciences Library, NYU Grossman School of Medicine, NYU Langone Health, New York, NY; 4 c.vandervleuten@maastrichtuniversity.nl, Professor, Department of Educational Development and Research, School of Health Professions Education, Faculty of Health, Medicine and Life Sciences, Maastricht University, Maastricht, the Netherlands; 5 anique.debruin@maastrichtuniversity.nl, Professor, Department of Educational Development and Research, School of Health Professions Education, Faculty of Health, Medicine and Life Sciences, Maastricht University, Maastricht, The Netherlands; 6 akalet@mcw.edu, Professor, Medical College of Wisconsin, Wauwatosa, WI

**Keywords:** Evidence Based Practice, Competency-Based Assessment, Medical Students, Evidence-Based Librarianship

## Abstract

**Objective:**

We expect medical students to be able to apply evidence-based medicine (EBM) skills in the context of the clinical care of patients. Previous assessments of this domain have primarily utilized decontextualized knowledge tests, which provide limited insights into students' understanding of EBM skills in the context of patient care. New performance-based EBM competence assessments using Objective Structured Clinical Examinations (OSCEs) are being developed and tested. Understanding how students experience and interact with a simulation-based assessment of EBM competence would enable us to improve the modality.

**Methods:**

We recruited 13 graduating medical students from one medical school who had recently completed an immersive multi station readiness-for-residency OSCE (Night onCall) which included a case-based EBM assessment. We conducted individual interviews to explore their perceptions of participating in this OSCE as a method of EBM assessment. The interviews were transcribed, coded, and analyzed using Dedoose by three health science librarians.

**Results:**

Students discussed their experience and perceptions in six main areas: connection to clinical practice, curricular timing and content coverage, feedback, station instructions, awareness of their own limitations, and an OSCE as a format for assessing EBM.

**Conclusion:**

Medical students appreciated the EBM OSCE because it enhanced their learning about how to integrate EBM into clinical practice. They proposed implementing multiple such opportunities throughout medical school because it would improve their competence and provide highly impactful opportunities to build toward EBM mastery. They endorsed that this would be well-accepted by medical students.

## INTRODUCTION

Evidence-based medicine (EBM) is an essential competency for practicing physicians [1, 2]. To engage in EBM, medical students must formulate focused, structured questions, search for related evidence, critically appraise and integrate this evidence with their own clinical experience and patient values [3, 4]. However, medical student training and assessment in EBM is often inconsistent and focused primarily on written assessments of knowledge [5-10]. The three primary validated assessments are the Berlin Questionnaire (multiple choice), the Fresno Test (essay), and the ACE Tool (true/false) [7, 9, 10]. While they each have their own strengths and weaknesses, a common weakness is that these validated assessments do not include any observation of applied behaviors. In their 2011 paper from the Sicily Conference, Tilson et al provide the CREATE Framework for classifying different EBM assessment tools [11]. The CREATE Framework identifies what type of testing is better for what level the assessment is aimed at: knowledge, skill, or behavior. Cognitive testing like the Berlin Questionnaire or the ACE Tool are best at assessing knowledge. Performance assessment, like the Fresno Test are best at assessing skill. Activity monitoring, on the other hand, is best at assessing competency in performing a behavior.

Strategies to build authentic, contextualized EBM competency assessments that utilize activity monitoring are emerging [11, 12]. EBM Objective Structured Clinical Evaluations (OSCEs) are one method being used to both assess competence and provide students feedback to enable development of EBM skill mastery [5, 13-15]. In 1999, the first two EBM OSCEs reported in the literature each focused only on one specific step within EBM: one on MEDLINE search creation and the other on critical appraisal of abstracts [16, 17]. While these demonstrated that the individual behaviors could be assessed in this format, they did not approximate real-world EBM practice. The following EBM OSCE efforts in the early 2000s moved the needle more, with one OSCE including multiple steps of EBM and the other focusing on critical appraisal [18-20]. Still both presented test environments that did not simulate the real world and did not have students ask questions, search for, identify, appraise, and apply the evidence they found in their own search. EBM OSCEs from 2009 and forward continued to move the needle with inclusion of all of the steps of EBM [15, 21, 22]. These three efforts used different approaches, but all found success in utilizing this format to effectively assess EBM competence.

However robust these efforts have been so far, none of them included the views of the learners in their efforts to assess effectiveness of the station – only if enough reliable metrics were generated. Also of note, only Burrows and Tylman's 1999 EBM OSCE explicitly included librarians, often the primary teachers of EBM content [17, 23]. Despite this progress, we still have a limited understanding of how medical students experience EBM OSCE stations. Understanding students' feedback and preferences and incorporating these perceptions into improving EBM competency assessments will improve the stations, allowing us to make them more practical and actionable to enhance student lifelong learning and not just be an assessment of one point in time. This study aims to explore the views and preferences of medical students taking an EBM OSCE in order to describe factors that lead to a successful experience that motivates students to further refine their competence and informs future development and refinement of EBM OSCEs as an assessment strategy.

## Methods

### Data Collection

In April 2022, all 107 graduating medical students in the NYU Grossman School of Medicine Class of 2022 were recruited via email to participate in semi-structured interviews about their experiences participating in an EBM OSCE, Night onCall. We offered each participants a $25.00 incentive, from the 2022 Medical Library Association (MLA) Research, Development, and Demonstration Project Grant. To be eligible, graduating students must have already completed the Night onCall OSCE. Recruitment continued until new data points no longer appeared in the interview responses to the semi-structured questions. Responses began to be primarily repetitive starting around the 6th interview. The authors continued interviewing until no new data points were revealed in 4 consecutive interviews, indicating to the authors that saturation had been reached [24]. Out of the 107 graduating medical students recruited, 13 were interviewed. Following interviews, all data was anonymized and no further data was collected about the interview participants.

The Night onCall EBM station was part of an immersive multi-station readiness-for-residency OSCE [13]. The station followed a patient case and had students sit at a computer to begin the station. Initially they were prompted to enter their most pressing clinical question(s) from the patient they had just seen, then on the next screen they were given an important clinical question about their patient and asked to find evidence to answer the question and describe how they would care for the patient given the evidence they found. They were not specifically told to use any particular sources and were able to freely use the Internet in whatever way they wanted. A full description of the Night onCall OSCE can be found in the previously published paper [13].

All NYU Grossman School of Medicine medical students were required to participate in this OSCE to receive formative feedback that might help guide their self-development as incoming residents. While these students had participated in many OSCEs throughout medical school, this was the first time in an EBM OSCE for all students. This group of medical students all received the same training on EBM throughout medical school. Their training consisted of a scaffolded six-month long curriculum in their first year, focusing on the first three steps of EBM: Ask, Acquire, and Appraise. This curriculum was followed by journal clubs and self-guided learning in clerkships where they practiced these steps and learned more about the final two steps: Applying evidence to patient care and Assessing their own performance.

We chose to conduct semi-structured medical student interviews about their experiences participating in this OSCE because this method enabled us to explore individual perceptions about the experience in detail, and generate extensive guidance to improve the impact of this activity. NYU Grossman School of Medicine IRB review led to the determination that this activity was educational quality improvement and thus exempt from full IRB review and approval. However, we obtained informed consent from participants and anonymized data prior to analysis to ensure participant confidentiality.

An iterative process was used to develop the semi-structured interview guide. Questions and prompts were initially developed based on a literature review and refined in discussion with co-authors to focus on the following key topics: student performance, feedback preferences, station logistics, connection to curriculum, connection to real-life behaviors, and other perceptions about assessment of EBM competency. See appendix 1 for the semi-structured interview guide.

One study author (JN) conducted these semi-structured 30-minute interviews using the question guide via Zoom and audio recorded and transcribed using the automatic transcription feature. Three authors (JN, JM, and CP) verified and corrected transcripts using the audio recordings. The interviewer (JN) also kept field notes during the interviews to aid in editing and verifying transcripts. Following verification, the transcripts were anonymized.

### Data Analysis

Analysis of the interview transcripts followed the stages in reflexive thematic analysis as defined and revised by Braun and Clarke [25-27]. This approach was used because of its flexibility and interpretive approach to understanding and describing patterns and meaning. A realist approach was used in this analysis, which attempts to identify themes that portray truths about the data. In a realist view, the data is assumed to reflect the true experiences and perceptions of the participant. The goal of the authors in utilizing a realist approach was to observe the data as participants' reported it, without trying to interpret the underlying social or cultural influences. Ultimately with a goal of presenting an authentic analysis of how participants experienced and felt about the OSCE.

Initially, three study authors (JN, JM, and CP) independently read all transcripts and developed an initial round of codes. Following this familiarization, they met to compare and discuss codes and insights so far. The same authors then progressed to independent deductive coding using an agreed upon set of 21 codes they developed jointly during the familiarization process. See Table 1 for code structure and frequency of use. In addition to the base codes, authors progressed into coding with an openness to changing, adding, or not using codes as they continued to understand the material. Each transcript was blind double-coded using the agreed upon codes and sections could be given multiple codes as appropriate. Because each author was coding independently, memoing was used to ensure a richer discussion of codes and themes later. Memoing, in this instance, refers to making notes on why you coded something a certain way or thoughts you had about potential meanings or clarifications. Table 1 displays the frequency of use of each code and the total number of codes applied across all interview transcripts.

**Table 1:** Code Frequency Distributions

Primary Codes and Subcodes	Frequency of Use	Primary Code Category Totals
Challenges in Completion	16	119
Time Constraints	32	
PICO Questions	28	
Station Instructions	43	
Perceived Performance	4	106
Overall Performance	26	
Asking a Question Performance	25	
Searching Database Performance	21	
Named a Resource	28	
Named a Mobile Device	2	
Station Expectations	14	75
Self-Performance Expectations	14	
Assessment Expectations	47	
Future Recommendations	62	62
Feedback to Students	24	56
Individualized Written Feedback	13	
Best Practice Video Feedback	19	
Timing/Frequency of EBM OSCE	52	52
Connection to Curriculum	46	46
EBM Behavior in Clinic vs OSCE	43	43
Impact of Participating	28	28
All Codes	587	

After the processes of familiarization, generation of codes, and deductive coding, the authors then moved into constructing themes. To achieve this, the authors extracted data on frequencies of code use and code overlap, using the code overlap as the primary clusters of meaning. This data was used by JN, JM, and CP independently to reflect on the meanings found in the data. Finally, through rounds of discussion, they defined and revised themes. In moving from codes to clusters of meaning to themes, JN, JM, and CP sought to describe what was important to the students' in their interview responses. As an example of this process, authors could take a comment that was coded as connection to curriculum, challenges in completion, and future recommendations and interpret it under the theme of recommending integration of EBM OSCEs earlier. In one comment a student might reflect on all three and draw a conclusion, while another student may only make one of those comments at a time during the interview.

Despite the interview guide being structured around three primary topics, the authors found six primary themes that frequently occurred in the students' answers. The realist approach continued to be utilized in this stage of thematic analysis, attempting to reflect the true observations and reality of the participants. The frequency of these clusters of meaning, both across students and within each student, helped solidify for the authors that these were the primary themes to highlight as findings. These six themes were then reviewed by all authors, bringing their expertise in medical education and assessment into the conversation. The Dedoose Desktop App, version 9.0.54 (SocioCultural Research Consultants, LLC, Los Angeles, CA, USA) was used to store and code the transcripts and generate data for thematic analysis. For a reflexivity statement on our qualitative thematic analysis, see Appendix 2.

## RESULTS

A total of 13 medical students participated in individual interviews following their experience participating in Night on Call. All participants had the same medical school curriculum and completed this OSCE 2–3 months prior to graduation.

### Thematic Analysis

Six primary themes emerged that highlight medical student experiences and preferences in participating in an EBM OSCE station.

**Theme 1: EBM has a clear connection to clinical practice.**

Students appreciated that they were being tested and receiving feedback on a competence they knew was important to clinical practice. They explained that their performance in this OSCE was not always reflective of how they would do the same thing in an actual clinical setting. In practice, they report being able to use the apps and bookmarked websites on their phones that they know and trust, as opposed to always having to open new tabs and begin searches from scratch. At the same time, they reported that participating in this OSCE helped make it clear to them when and how to use EBM in a more clinically effective way.

*“I think when we learn these things we're like, “oh yeah, that's definitely gonna be important” but you don't realize how important until you really have both been in situations where you need to answer a question and are imminently faced - like it feels more real now that I'm going to be alone at times, and not have access to people to just ask questions to.” (S4)*

*“I've seen people do this even in clinical practice in my rotations, if there is a clinical question that comes up, it's important to be able to identify the question in a timely manner, and use the right terminology to find the answers that you need, and also be able to find the resources to answer your questions.” (S11)*

**Theme 2: Integration of EBM OSCEs should occur earlier in the curriculum to allow for practice and mastery.**

Students stressed that there was too big of a gap between when they were taught this content and the timing of this station. Three specific recommendations surfaced multiple times. First, they suggested we introduce an EBM OSCE station near the beginning of clerkships to help set clear standards for performance and time to practice during clerkships. Second, students specifically requested that we introduce an EBM OSCE station during Sub-Internships when they are most actively practicing this skill set. And third, they asked that we expand the clinical areas covered in the EBM OSCE to reflect specialties beyond primary care adult medicine.

*“I feel like this would be almost something that would be useful, like before your clerkship year or during your clerkship year. I thought a lot of this stuff that we did would actually be really useful to have during your sub-I because you do have some of these responsibilities. This is when you're learning all of these responsibilities, learning more to be like an intern which I guess is the point of all these stations.” (S6)*

*“if the purpose is for us to be able to implement something new or to grow. I think that having multiple chances to take a shot at it is always helpful. So I think it would have been helpful. I actually think it probably could have been helpful to have that before clerkship year. And just maybe have like gotten feedback on: Okay, this is what you did and here's another thing that you can do for next time. And then had the chance to get reassessed after clerkships to see if we've been able to incorporate some of the feedback.” (S7)*

**Theme 3: Feedback is important to build and solidify competence.**

Students all indicated that timely feedback was essential to the success of learning from their OSCE experience but they differed on the type of feedback they preferred. Students who reported feeling they did well in the Night onCall EBM OSCE station expressed a desire to see a video of a best-practice model answer to the station. Those who felt like they had room to improve wanted actionable individual feedback tailored to their own performance.

*“if it's individualized feedback I think I'm more likely to remember at least one of those pointers and incorporate it next time versus in the more generalized feedback. I think, as a student as someone with a busy schedule there's always the chance that I don't even watch the 2 min video.” (S7)*

*“I think a model is great - I don't think it needs to be individualized. I think that would be a lot of work on all of your ends and then we're all intelligent individuals and can look at what our search was like if you provide us what we searched in retrospect, and then what a model search would be like, we can all tailor our searches from there. “ (S8)*

**Theme 4: Low self-confidence in ability and perceived skill to effectively and efficiently perform searches.**

Students noted two main concerns regarding how well the EBM competency OSCE reflected their EBM proficiency-time on task and technical proficiency. Some students believed that the time limit prevented them from demonstrating their full ability. In particular, students who reported having a background in conducting research noted that they were used to having more time to do searches and interpret and apply results. Other students reported a lack of confidence in their technical ability to perform searches using standard tools such as PubMed and attributed this low self-confidence to having had little coaching on these skills during the clinical years.

*“I'm just not that adept at like…I can figure it out in PubMed, but not that quick. So I was like, there's no time, I must find the answer from a quick search.” (S6)*

*“when I do a literature search I try to take my time with it, and given the time constraints, I had to arbitrarily pick data or pick searches that I wouldn't normally do” (S2)*

**Theme 5: OSCE stations on novel tasks are cognitively challenging.**

While all the medical students reported they were able to adequately complete the station, they did express surprise at encountering a novel task in simulation so close to graduation. Students provided detailed recommendations for improving station instructions and they expressed a desire to have this type of station introduced earlier in their medical school experience. They believed this would help students focus their attention on completing the tasks rather than understanding instructions.

*“I remember for me feeling like: Well, I spent the whole first three minutes figuring out what I was supposed to do and then I have like six minutes to do it.” (S5)*

*“I never read instructions well when I'm in the OSCE situation. I've always found that oral instructions at the beginning, and then reminders at the station work best for me.” (S4)*

*“a lot of that OSCE was just very different than the OSCEs that we've had in the past. And so I think, at least for me, a lot of it was spent just trying to understand the system.” (S5)*

**Theme 6: A simulated clinical context, like an OSCE, is an impactful way to practice and gain feedback on EBM skills.**

Students reported feeling that an EBM OSCE aligned well with simulations of other clinical competencies. This helped them realize that EBM skills are clinical skills they will be responsible for practicing. They also pointed out the disconnect between what they are taught in classes, such as PICO, and real-world practice. All of the students requested more chances to practice and get feedback on this important set of skills.

*“I think the main takeaway – I feel like there's always this divide between formulating a PICO question and then maybe doing a literature search or something like that, what we might do research-wise, and then what we actually do in the hospital. And I feel like in the hospital we're usually just going by protocol or going by what we see other people doing rather than actually sitting down ourselves and doing a literature search. And so I feel like this sort of provided a scenario when we could practice creating this link between a clinical situation and then that sitting down and literature-searching type of situation.” (S1)*

*“My takeaway was that I still have not mastered that skill! I could definitely get better at quickly finding those answers still, or I guess finding them at all, because I didn't really find a great answer. So I guess I probably need both more practice and more instruction in the PubMed part.” (S4)*

## DISCUSSION

Medical students that participated in these interviews expressed sincere enthusiasm about the opportunity to practice and receive feedback on their EBM skills, especially because it was integrated into a comprehensive clinically authentic simulation. Within many medical schools' curricula, this content is often taught exclusively in the pre-clinical years. This can create a disconnect where students learn a theoretical framework, like PICO, but never learn to apply that framework in practice or learn in practice novel ways of approaching EBM that do not reflect what is taught. Situating this assessment integrated with other clinical skills helps emphasize and deepen the connection to clinical practice [28, 29].

Having recognized the clinical importance of practicing EBM, near-graduating students expressed a desire to have had more frequent and earlier EBM OSCE stations. They recommended having opportunities for this assessment throughout their clinical years, at least: one at the beginning of clerkships, and one during their sub-internships. Students would be better able to practice EBM clinically and master this set of skills prior to graduation if they were given multiple opportunities to be assessed and receive feedback. Introducing this type of assessment earlier would also serve to familiarize students with this type of competency assessment, reducing the extraneous cognitive load associated with figuring out the tasks under time restraint, enabling them to more accurately demonstrate their ability to do the components of the task germane to competency in authentic clinical settings [30, 31].

Students also commented that they would prefer the EBM OSCE station to feature subject matter related to their chosen specialty. By aligning and developing new EBM OSCE stations in partnership with clerkship directors, we could meet the goal of more frequent opportunities for practice, broaden the subjects covered, and tailor these assessments so that, when appropriate they are relevant to the student's chosen career path.

Feedback was seen as a key component of learning from this station. However, students differ about what kind of feedback they preferred. Students who self-identified as more experienced in research wanted to be able to walk away with a video model of best practice that they could keep as a reference. Students who self-identified as weaker at this skill set were hoping for individually tailored actionable feedback based on their specific performance. This is further supported by research on the expertise reversal effect in cognitive load theory [32]. The expertise reversal effect is seen when overly detailed feedback actually reverses the expertise of more advanced learners, making them question what they already know. For novice learners, worked examples with step-by-step instructions and individual guidance are helpful for learning. For more advanced learners, fully worked examples with individual guidance become redundant and can lead to expertise reversal and worse performance [33-35]. Taking this into account, using a fading guidance strategy in providing feedback will be more effective for the learners [36]. These recommendations align with introducing multiple EBM OSCEs throughout medical schools. The OSCEs taking place earlier in the curriculum could have tailored feedback, and as students become more experienced and skilled they could receive less guidance.

Students did point out some frustrations and problems with this station. Their difficulties hinged on their self-reported kind of experience. Students with a heavy research background were more used to having a lot of time to search for and synthesize evidence. These students struggled with the time limit. Students with limited research experience struggled with the best ways to search for evidence. These students felt that they could not excel in this station because of their limited technical skills. Both time limitation and technical skill are expected and desirable difficulties that force students to critically examine their habits and assumptions. Despite students reporting these as frustrations, they also reflected on them as learning opportunities. This self-reflection is a critical part of facilitating transformative learning [37]. Knowing this, we can provide feedback and advice tailored to student experience and focused on raising student awareness of their proficiency or lack thereof.

Operationally, students understood the instructions and were able to complete the station. However, when pressed for suggestions, they suggested a variety of improvements ranging from announcing the instructions aloud to reviewing the instructions for each station before the OSCE begins, to allowing a preview of the instructions the week before. The common theme in their suggestions was that they were surprised to be experiencing a station that was new to them so close to graduation. Cognitive load theory is important to consider here. Cognitive load theory explains how, in performing tasks like EBM with many elements and a high degree of interactivity, the intrinsic load is high. This becomes problematic if the extraneous load of processing new instructions is also high, leading to weaker performance due to the additive cognitive load [30, 32]. Instead of changing the instruction delivery, this discomfort could be addressed by having a station in this format occur throughout the curriculum, thus decreasing the extraneous load and allowing students more time to focus on the task at hand [38].

## LIMITATIONS

There are several possible limitations for our study. Our volunteer sample of medical students is a snapshot of graduating medical students from one medical school. The experiences, needs, and preferences of students could differ depending on their unique backgrounds and medical school curricula. Qualitative data can be interpreted in many ways. The three study authors who coded and analyzed the data are practicing medical librarians who teach and assess EBM. We approached this study with a desire to maximize the trustworthiness of the findings through reflexivity and by incorporating memoing, field notes, independent descriptive coding, iterative discussions, and adjudication of disagreements in order to come to shared, constructed interpretations. Our process did not include activities such as member checking to enhance validity. Instead, we felt that our expertise and prolonged exposure in the field when combined with thematic saturation of the data were sufficient.

## CONCLUSION

Medical students appreciated having the opportunity to take an EBM OSCE. Students liked this OSCE and saw a great value in it as a learning experience. They recognized that the OSCE also helped them solidify how EBM integrates into clinical practice. Their experience could be improved by having multiple opportunities throughout medical school to participate and receive feedback on their performance in this OSCE.

While this study involved a limited sample of 13 participants, using qualitative research methods allowed us to go deeper into experiences and perceptions and find more nuanced perspectives. That our findings align with established research on competency-based assessment, such as the importance of formative feedback and multiple assessments over time, strengthens our confidence in the findings [39]. However, further research with larger and more diverse cohorts of students from other types and sizes of medical schools would help generalize these results and allow for exploration of themes across institutions.

From this study, we are confident that implementing a series of EBM OSCEs would be well-accepted by medical students and a powerful opportunity to both assess competence and build mastery. Future research on a longitudinal series of EBM OSCEs could investigate the effectiveness of different types of feedback and explore the impact of the interprofessional nature of librarian-led EBM OSCEs impact on both medical education processes and learning outcomes. Overall, our findings indicate that librarian-led OSCEs are an acceptable and useful method for advancing competency-based assessment of EBM.

## Data Availability

Data associated with this article are available in Zenodo at: https://doi.org/10.5281/zenodo.11390561.
