# Supplementary material for: “I still have not mastered that skill!” Medical student perspectives on a simulation-based evidence-based medicine competency assessment
Source: J Med Libr Assoc. 2025 Apr 18;113(2):168–76. doi: 10.5195/jmla.2025.2023 (PMC12058340; doi:10.5195/jmla.2025.2023)
Supplement: Supplementary file 1 — Appendix A [file jmla-113-2-168-s01.docx]

Evidence-Based Medicine OSCE: Medical Student Perspectives

Semi-Structured Interview Guide developed by [redacted]

Station Goals:

Please tell me what you recall about the clinical question station during Night-OnCall?

What do you think you did well in this station?

What were some of the difficulties you had with the station?

What would you say you personally took away from this case?

Did this case assess skills and knowledge you had learned prior to the OSCE?

Feedback:

What feedback were you given?

-What did you think about the feedback you received on this station?

-Can you recall any specifics of the feedback? How was it delivered?

Knowing you would be evaluated on your performance, did it change how you performed your search and answered the question?

What did you think you were being assessed on? [written answer vs. process]

Do you have any suggestions for improving the feedback and/or the feedback process? What would you like to have received feedback on? In what kind of format?

Logistics:

What did you think about the way this station worked?

What do you think about the timing of this station in the curriculum? Is getting feedback on this set of skills useful to you at this time? Would it help or be differently received earlier?

Do you have any suggestions for improving this case and/or this station?

Any additional thoughts about the library and how we might be able to help or support medical students more?
