# Supplementary material for: “I still have not mastered that skill!” Medical student perspectives on a simulation-based evidence-based medicine competency assessment
Source: J Med Libr Assoc. 2025 Apr 18;113(2):168–76. doi: 10.5195/jmla.2025.2023 (PMC12058340; doi:10.5195/jmla.2025.2023)
Supplement: Supplementary file 2 — Appendix B [file jmla-113-2-168-s02.docx]

Appendix 2

Reflexivity Statement for the JMLA article: **"I still have not mastered that skill!" Medical Student Perspectives on a Simulation-Based Evidence-Based Medicine Competency Assessment**

This reflexivity statement is intended to enhance transparency and credibility of our qualitative thematic analysis. The backgrounds and experiences of the authors potentially have an influence on both the research process and findings. This is important context to understand when reading, appraising, and interpreting the results of this study. We acknowledge that the conduct and results of this study are co-constructed among the authors and the participants. The interviewer (JN) has a master’s degree in library and information science and a master’s degree in public health. He has been working as a health sciences librarian both teaching and assessing EBM for 20 years. He was never the EBM instructor for this group of graduating students, yet was familiar with their full curriculum and the design of the entire OSCE, not limited to the EBM station. This background allowed him to connect with the students not as one of their professors, but as an interested party with whom they could feel comfortable candidly sharing their experiences. The interviewer (JN), transcribers (JN, JM, CP), and coders (JN, JM, CP) are all practicing health sciences librarians actively engaged in teaching and assessment of EBM.
